# Bacterial DNA and osteoarthritis in dogs with patellar luxation and cranial cruciate ligament rupture

**DOI:** 10.14202/vetworld.2023.2049-2054

**Published:** 2023-10-07

**Authors:** Sirun Tuek-Um, Sarawut Yangtara, Win Surachetpong, Sarawan Kaewmongkol, Gunn Kaewmongkol, Naris Thengchaisri

**Affiliations:** 1 Surgery Unit, Kasetsart University Veterinary Teaching Hospital, Bangkhen campus, Bangkok 10900, Thailand; 2 Department of Companion Animal Clinical Sciences, Faculty of Veterinary Medicine, Kasetsart University, Bangkok 10900, Thailand; 3 Department of Veterinary Microbiology and Immunology, Faculty of Veterinary Medicine, Kasetsart University, Bangkok 10900, Thailand; 4 Department of Veterinary Nursing, Faculty of Veterinary Technology, Kasetsart University, Bangkok, Thailand

**Keywords:** bacterial DNA, cranial cruciate ligament, osteoarthritis, stifle

## Abstract

**Background and Aim:**

The association between bacterial DNA in stifle joints, including those with cranial cruciate ligament rupture (CCLR) and medial patellar luxation (MPL), and osteoarthritis in dogs remains elusive. This study investigated the potential association between the detection of bacterial DNA and osteoarthritis in dogs using a broad-range polymerase chain reaction technique targeting the 16S ribosomal RNA gene.

**Materials and Methods:**

Synovial fluid (35 samples) and knee tissue samples (32 samples) were obtained from 35 dogs diagnosed with CCLR (n = 20; 11 males and nine females) or MPL (n = 15; five males and 10 females) who underwent a surgical operation between October 2014 and April 2015.

**Results:**

Dogs with CCLR had a higher average osteoarthritis score than those with MPL (2.0 ± 0.9 vs. 0.5 ± 0.9; p = 0.005). Bacterial DNA was detected in the stifle joints of 60.71% of dogs with MPL. *Pelomonas* spp. (25.00%), *Halomonas* spp. (17.86%), and 5 other species (17.86%) were the most frequently identified bacteria. Bacterial DNA was detected in 41.03% of dogs with CCLR. *Pelomonas* spp. (15.38%), *Sphingomonas* spp. (10.26%), *Halomonas* spp. (5.13%), and 4 other species (10.26%) were the most frequently identified bacteria. No significant difference was observed in the prevalence of bacterial DNA obtained from tissue samples (46.88%) or joint fluid samples (51.43%). The presence of bacterial DNA was not associated with the type of knee injury (MPL or CCLR; p = 1.000). There was a higher prevalence of bacterial DNA in samples from dogs with moderate-to-severe osteoarthritis (94.44%) than in those with minimal osteoarthritis (41.18%), and a significant association between the presence of bacterial DNA and moderate-to-severe osteoarthritis was identified (p < 0.01).

**Conclusion:**

Dogs with moderate-to-severe osteoarthritis were more likely to have bacterial DNA in their stifle joints than those with no or minimal osteoarthritis. These findings provide valuable insight into the potential role of bacterial DNA in joint tissue or joint fluid and the development of osteoarthritis in dogs.

## Introduction

Medial patellar luxation (MPL) and cranial cruciate ligament rupture (CCLR), resulting in inflammation, lameness, and osteoarthritis, are the leading causes of leg pain in dogs [1, 2]. These conditions primarily affect the stifle joint and are most commonly observed during the stance and swing phases of movement [3, 4]. Osteoarthritis can decrease a pet’s quality of life due to chronic pain and joint degeneration [5]. These conditions arise from bone inflammation and structural abnormalities [6]. If left untreated, prolonged MPL and CCLR can result in increased contact between the femur and tibia resulting in osteoarthritis [7].

The previous studies by Heim *et al*. [8], and Gibb and Hadjiargyrou [9] have identified environmental bacteria that can cause joint infections in humans and dogs. Various pathogenic bacterial DNA, including *Borrelia burgdorferi* and *Chlamydia trachomatis*, among others, have been identified in arthritic joints indicating movement of bacteria into the joints [10]. Bacterial DNA is frequently detected in the knee joint [11]. In dogs with arthritis, the synovium is infiltrated with mixed groups of inflammatory cells, including B and T lymphocytes and macrophages. Notably, the amount of bacterial DNA found in canine joints is highest in the winter and spring and lowest in the summer. This suggests that environmental factors may influence the spread of bacteria to the joints [12]. However, it remains unclear whether the detection of bacterial DNA in joints correlates with the development of joint inflammation and osteoarthritis [13]. The development of primers specifically targeting bacterial DNA in the 16S ribosomal RNA (rRNA) region is valuable for identifying bacterial DNA from joint tissue or samples [14]. The connection between the bacterial species and the severity of osteoarthritis is not well established, and it is uncertain whether the presence of specific bacterial DNA is linked to MPL or CCLR. If specific bacteria are detected, their presence may indicate a potential risk for the development of osteoarthritis in companion animals [15, 16].

This study aimed to estimate the prevalence of bacterial pathogens in synovial fluid and tissue of the canine knee using DNA detection methods. The association between the presence of bacterial DNA and stifle joint disorders (MPL and CCLR), as well as osteoarthritis severity, was also evaluated. The data provide important information for designing future studies on animals at risk for joint degeneration and may also serve as a useful diagnostic tool for canine patients.

## Materials and Methods

### Ethical approval

The Kasetsart University Institutional Animal Care and Use Committee granted approval for all procedures under the reference number #U1-00500-2558. We obtained written consent from every dog owner, ensuring that all procedures adhered to the standards set by the Kasetsart University Institutional Animal Care and Use.

### Study period and location

The study was conducted from October 2014 to December 2015 in dogs with either MPL or CCLR visiting Orthopedic unit at Kasetsart University Veterinary Teaching Hospital, Bangkhen campus.

### Animals

This study included 35 dogs, 15 of which had MPL (five males and 10 females) and 20 of which had CCLR (11 males and nine females). Among the 20 dogs with CCLR, there were five Poodles, five crossbreeds, four Chihuahuas, two Yorkshire Terriers, two Pomeranians, one Beagle, and one Shih Tzu. In the 15 cases of MPL, there were six Pomeranians, four Chihuahuas, two Poodles, two crossbreeds, and one Shih Tzu.

**Figure-1:**
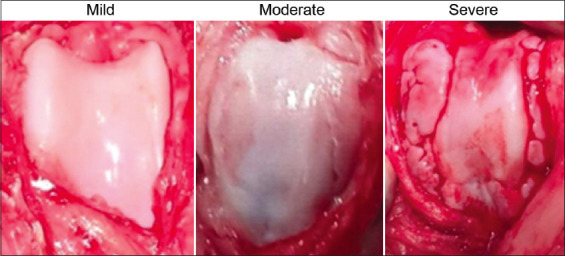
Severity level of knee joint degeneration in dogs with cranial cruciate ligament rupture or medial patellar luxation.

This study used a body condition score scale with five levels: (1) Emaciated; (2) thin; (3) ideal; (4) heavy; and (5) grossly obese. In addition, an osteoarthritis score scale was used, which consisted of three levels: (1) Mild (dogs showing pain in the limb but no visible signs of disease of the joint); (2) moderate (dogs showing pain and visible signs of disease such as lesions of the joint); and (3) severe (dogs showing severe pain and visible signs of disease such as severe lesions and bone deformities of the joint) (Figure-1).

### Polymerase chain reaction (PCR) analysis and DNA sequencing

DNA extraction from synovial fluid and tissue of the knee for PCR was performed using the equipment and methods recommended by the E.Z.N.A.® tissue DNA Kit (Omega Bio-tek, Inc., Norcross, GA). Once the DNA was extracted, the prevalence of DNA from various bacterial pathogens was determined using 16S rRNA nested PCR, which covers a wide range of bacterial species [17]. The 16S recombinant (rDNA) primers for the primary PCR consisted of V1-F and V9-R. The primary PCR was performed using 1 μL of DNA in 25 μL of the solution containing 1 × PCR buffer, 2 mM MgCl_2_, 0.2 mM deoxynucleotide triphosphates (dNTPs), 1 μM of each primer, and 0.04 U/μL Taq DNA polymerase. The primary cycle consisted of a pre-PCR step at 95°C for 5 min, followed by 40 cycles at 95°C for 60 s, 50°C for 60 s, an extension at 72°C for 90 s, with a final extension at 72°C for 10 min. The length of the primary PCR product was approximately 1400 bp. The PCR primers for the secondary PCR consisted of V3-F and V6-R [17]. Secondary PCR was performed using 1 μL of DNA in 25 μL of the solution containing 1× PCR buffer, 2 mM MgCl_2_, 0.2 mM dNTPs, 1 μM of each primer, and 0.04 U/μL taq DNA polymerase. The secondary cycle consisted of a pre-PCR step at 95°C for 5 min, followed by 45 cycles at 95°C for 60 s, 55°C for 45 s, an extension at 72°C for 45 s, with a final extension at 72°C for 10 min. The length of the secondary PCR product was approximately 700 bp. All PCR products were purified from agarose gel slices using a DNA purification kit (UltraCleanTM 15 DNA Purification Kit, MO BIO Laboratories Inc., Carlsbad, CA). Sequencing was performed using a terminator cycle sequencing kit (ABI PrismTM Terminator Cycle Sequencing Kit, Applied Biosystems, Foster City, CA) in an Applied Biosystems 3730 DNA analyzer following the manufacturer’s instructions. The 16S rDNA sequences were analyzed using the basic local alignment search tool. The PCR primers are listed in Table-1 [17].

**Table-1:** Primers and sequences used in this study.

Primers	Organism	Locus	Nucleotide sequences	Reference
V1-F	Broad range	16S	AGAGTTTGATCCTGGCTCAG	[17]
V9-R	bacteria	rRNA	GNTACCTTGTTACGACTT	
V3-F			ACTCCTACGGGAGGCAGCAG	
V6-R			CGACAGCCATGCANCACCT	

### Statistical analysis

Data were analyzed using a one-way analysis of variance with the program Prism version 6 (GraphPad Software, Boston), and differences between groups were compared using Dunn’s multiple comparisons test. The association between bacterial DNA and stifle joint diseases (MPL and CCLR) and osteoarthritis severity was evaluated using Fisher’s exact test with STATA version 12.2. (StataCorp LLC, College Station, TX, USA) The significance level was set at p < 0.05.

## Results

**Figure-2:**
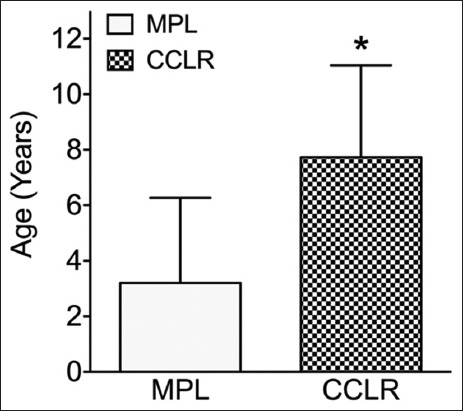
Average age of dogs with cranial cruciate ligament rupture (CCLR) and medial patellar luxation (MPL). Dogs with CCLR were significantly older than dogs with MPL.

**Figure-3:**
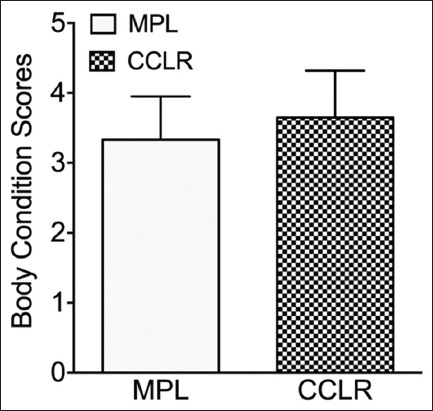
Body condition scores of dogs with cranial cruciate ligament rupture (CCLR) and medial patellar luxation (MPL).

**Figure-4:**
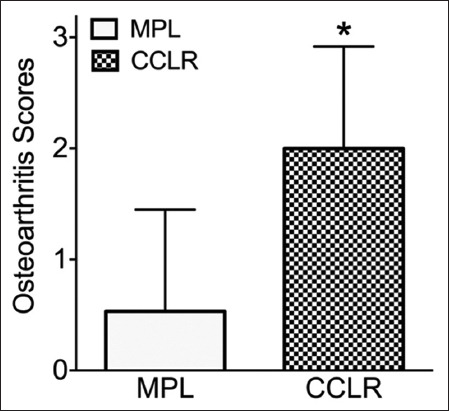
Osteoarthritis scores (on a scale of 0–3) of dogs with cranial cruciate ligament rupture (CCLR) and medial patellar luxation (MPL). Dogs with CCLR had higher average osteoarthritis scores than dogs with MPL.

The average age of dogs diagnosed with CCLR was significantly higher than that of dogs with MPL (7.7 ± 3.3 years vs. 3.2 ± 3.1 years; p = 0.008; Figure-2). However, there was no significant difference in body condition score between dogs with CCLR and those with MPL (3.3 ± 0.6 vs. 3.7 ± 0.7; p = 0.48; Figure-3). The severity of knee osteoarthritis was also evaluated, and dogs with CCLR had a significantly higher average score than those with MPL (2.0 ± 0.9 vs. 0.5 ± 0.9; p = 0.005; Figure-4).

The DNA of bacterial samples in synovial tissue (n = 32) and synovial fluid (n = 35) was analyzed in dogs with MPL and CCLR (Table-2). There was no significant difference in the prevalence of bacterial DNA between dogs with MPL (60.71%) and those with CCLR (41.03%; p = 0.141). The most commonly detected bacteria in dogs with MPL were *Pelomonas* spp. (25.00%), *Halomonas* spp. (17.86%), and 5 other species (17.86%) (Table-2). For dogs with CCLR, *Pelomonas* spp. (15.38%), *Sphingomonas* spp. (10.26%), *Halomonas* spp. (5.13%), and 4 other species (10.26%) were the most frequently identified bacteria, (Table-2). The overall prevalence of bacterial DNA in joint samples was highest for *Pelomonas* spp. (19.40%), *Halomonas* spp. (10.45%), and *Sphingomonas* spp. (7.46%) (Table-2). The prevalence of bacterial DNA identified from tissue samples (46.88%) and joint fluid samples (51.43%; p = 0.808) was comparable (Table-3).

**Table-2:** Prevalence of bacterial DNA in joint samples in dogs with cranial cruciate ligament rupture and medial patellar luxation.

Type of bacterial DNA	Medial patellar luxation (n = 28)		Cranial cruciate ligament rupture (n = 39)		Total (n = 67)	
		
	Positive (n)	% positive	Positive (n)	% positive	Positive (n)	% positive
*Sphingomonas*spp.	1	3.57	4	10.26	5	7.46
*Pelomonas*spp.	7	25.00	6	15.38	13	19.40
*Achromobacter*spp.	1	3.57	1	2.56	2	2.99
*Pseudomonas*spp.	0	0.00	1	2.56	1	1.49
*Moraxella*spp.	1	3.57	0	0.00	1	1.49
*Halomonas*spp.	5	17.86	2	5.13	7	10.45
*Corynebacterium*spp.	1	3.57	0	0.00	1	1.49
*Streptococcus*spp.	0	0.00	1	2.56	1	1.49
*Kocuria*spp.	1	3.57	0	0.00	1	1.49
*Propionibacterium*spp.	0	0.00	1	2.56	1	1.49
Total	17	60.71	16	41.03	33	49.25

**Table-3:** Prevalence of bacterial DNA in tissue or fluid samples in dogs with cranial cruciate ligament rupture and medial patellar luxation that underwent stifle surgery.

Type of bacterial DNA	Tissue sample (n = 32)		Joint fluid sample (n = 35)		Total (n = 67)	
		
	Positive (n)	% positive	Positive (n)	% positive	Positive (n)	% positive
*Sphingomonas*spp.	3	9.38	2	5.71	5	7.46
*Pelomonas*spp.	6	18.75	7	20.00	13	19.40
*Achromobacter*spp.	1	3.13	1	2.86	2	2.99
*Pseudomonas*spp.	1	3.13	0	0.00	1	1.49
*Moraxella*spp.	0	0.00	1	2.86	1	1.49
*Halomonas*spp.	3	9.38	4	11.43	7	10.45
*Corynebacterium*spp.	0	0.00	1	2.86	1	1.49
*Streptococcus*spp.	0	0.00	1	2.86	1	1.49
*Kocuria*spp.	1	3.13	0	0.00	1	1.49
*Propionibacterium*spp.	0	0.00	1	2.86	1	1.49
Total	15	46.88	18	51.43	33	49.25

There was no association between the presence of bacterial DNA and the type of knee injury (MPL or CCLR; p = 1.000). Bacterial DNA was more common in dogs with moderate-to-severe osteoarthritis (94.44%) than in those with minimal osteoarthritis (41.18%). Furthermore, there was a significant association between the presence of bacterial DNA and moderate-to-severe osteoarthritis (p < 0.01), as noted in Table-4.

**Table-4:** Association between type of knee injury or canine osteoarthritis severity and the presence of bacterial DNA in stifle joint samples.

Category	Negative for bacterial DNA		Positive for bacterial DNA	
	
	n	(%)	n	(%)
Surgical procedure				
Medial patellar luxation	5	33.33	10	66.67
Cranial cruciate ligament rupture	6	30.00	14	70.00
Total	11	31.43	24	68.57
Osteoarthritis score				
Normal to minimal (0–1)	10	58.82	7	41.18
Moderate to severe (2–3)	1	5.56	17	94.44**
Total	17	48.57	18	51.43

** p < 0.01 for test of association between osteoarthritis score and presence of bacterial DNA.

## Discussion

This study compared the prevalence of bacterial DNA in the stifle joint of dogs that underwent surgery for MPL or CCLR. The average age of dogs with CCLR was significantly higher than that of dogs with MPL. The two groups had no significant difference in their body condition scores, but dogs with CCLR had more severe knee osteoarthritis than dogs with MPL. The types of bacterial DNA found in synovial tissue and fluid were comparable, with *Pelomonas* spp. being the most common. The present study found no difference in the presence of bacterial DNA in synovial fluid and tissue between dogs with MPL (60.71%) and those with CCLR (41.03%; p = 0.141). However, dogs with moderate-to-severe osteoarthritis were more likely to have bacterial DNA than those with normal-to-minimal osteoarthritis.

The synovial fluid in joints, primarily composed of hyaluronic acid, is important for lubrication and cushioning [4, 18]. Inflammatory conditions such as MPL and CCLR can decrease the production of hyaluronic acid, weakening the ligament and further harming the joint [10]. Even surgical correction of these issues can impair joint stability and decrease the effectiveness of joint fluid. Moreover, a persistent, slowly progressing, bacterial infection might lead to substantial deterioration of the graft with a prolonged inflammatory setting. This could lead to additional compromises of the graft’s structural integrity [19]. Analyzing bacterial samples in synovial tissue and fluid can provide important insights into the underlying pathophysiology of these conditions. This study found bacterial DNA in the stifle joint in both CCLR and MPL cases. It is worth noting that the prevalence of bacterial DNA was higher in the stifle joint of dogs with severe osteoarthritis. Nonetheless, the previous study by Wang *et al*. [20] has found bacterial DNA in normal joints, suggesting that the presence of bacterial DNA may not be specifically associated with arthritis [21]. It is possible that the detected bacteria entered through blood flow to the synovial membrane [22, 23].

The previous studies by Prior and Arthurs [24] and Innes *et al*. [25] have suggested that certain environmental bacteria may be linked to joint infections in both humans and dogs [24], potentially indicating a connection between bacteria and the development of arthritis [25]. However, the relationship between the presence of bacterial DNA in joints and inflammation or osteoarthritis remains unclear [26]. This study found an association between the presence of bacterial DNA and the severity of osteoarthritis in dogs with common knee injuries [27]. A recent study found that bacterial DNA may indicate an increased risk of developing osteoarthritis in animals although specific bacteria were not identified [28]. In our study, the detection of *Pelomonas* spp., *Halomonas* spp., and *Sphingomonas* spp. in joint samples suggests that bacterial infections may play a role in developing CCLR and MPL [10, 19]. The association in this study between the presence of bacterial DNA and moderate-to-severe osteoarthritis further supports this hypothesis. The yield of synovial fluid cultures in dogs with suspected infectious arthritis is known to be low [29]. Reported sensitivity remains low [15], with false negatives being reported in a significant proportion of cases (20%–50% of dogs, 12%–43% of humans, and 23.7% of horses) with septic arthritis [13]. Further research is necessary to explore the impact of environmental factors on the development of knee conditions in dogs, and advanced techniques such as broad-range PCR can be used to detect bacterial DNA in synovial fluid and tissue as a predictive indicator for the development of osteoarthritis in dogs [17, 30].

This study has certain limitations. One was broad-range PCR with a universal primer that could not differentiate between viable and non-viable bacteria. It is important to note that while the 16S ribosomal RNA gene is highly conserved, it also has variable regions. In addition, we employed Sanger sequencing after the nested PCR protocol. Sanger sequencing only sequences one DNA fragment at a time, whereas next-generation sequencing (NGS) can sequence millions of fragments simultaneously per run. Therefore, future studies using NGS are necessary to identify bacterial DNA from joint samples.

## Conclusion

This study found that the presence of bacterial DNA is not specific to MPL and CCLR knee conditions in dogs. However, we identified a significant association between moderate-to-severe osteoarthritis and the presence of bacterial DNA in this study. Further research is needed to explore the relationship between bacterial DNA, joint inflammation, and osteoarthritis in dogs. Future studies should continue to explore the role of bacterial infections in the pathogenesis of these conditions and evaluate the efficacy of antibiotics and other anti-infective treatments in their management.

## Data Availability

The data that support the findings of this study are available from the corresponding author on a reasonable request.

## Authors’ Contributions

ST and NT: Conceptualization. ST, SY, SK, GK, and NT: Conducted the study. ST, SK, GK, and NT: Methodology. ST, WS, and NT: Data analysis and Writing-original draft preparation. ST, SY, WS, SK, GK, and NT: Writing-review and editing. All authors have read, reviewed, and approved the final manuscript.
